# Supplementary material for: Defining a Midgestational Window for In Utero Genome Editing of the Fetal Murine Cortex
Source: bioRxiv. 2026 May 1:2026.04.28.721509. Preprint. [Version 1] doi: 10.64898/2026.04.28.721509 (PMC13142468; doi:10.64898/2026.04.28.721509)
Supplement: Supplement 1 [file NIHPP2026.04.28.721509v1-supplement-1.pdf]

bioRxiv preprint doi: https://doi.org/10.64898/2026.04.28.721509; this version posted May 1, 2026. The copyright holder for this preprint (which was not certified by peer review) is the author/funder, who has granted bioRxiv a license to display the preprint in perpetuity. It is made available under aCC-BY 4.0 International license.

Figure S1: Barcoded AAV capsid screening at E15.5 reveals no enhanced CNS tropism over AAV9.

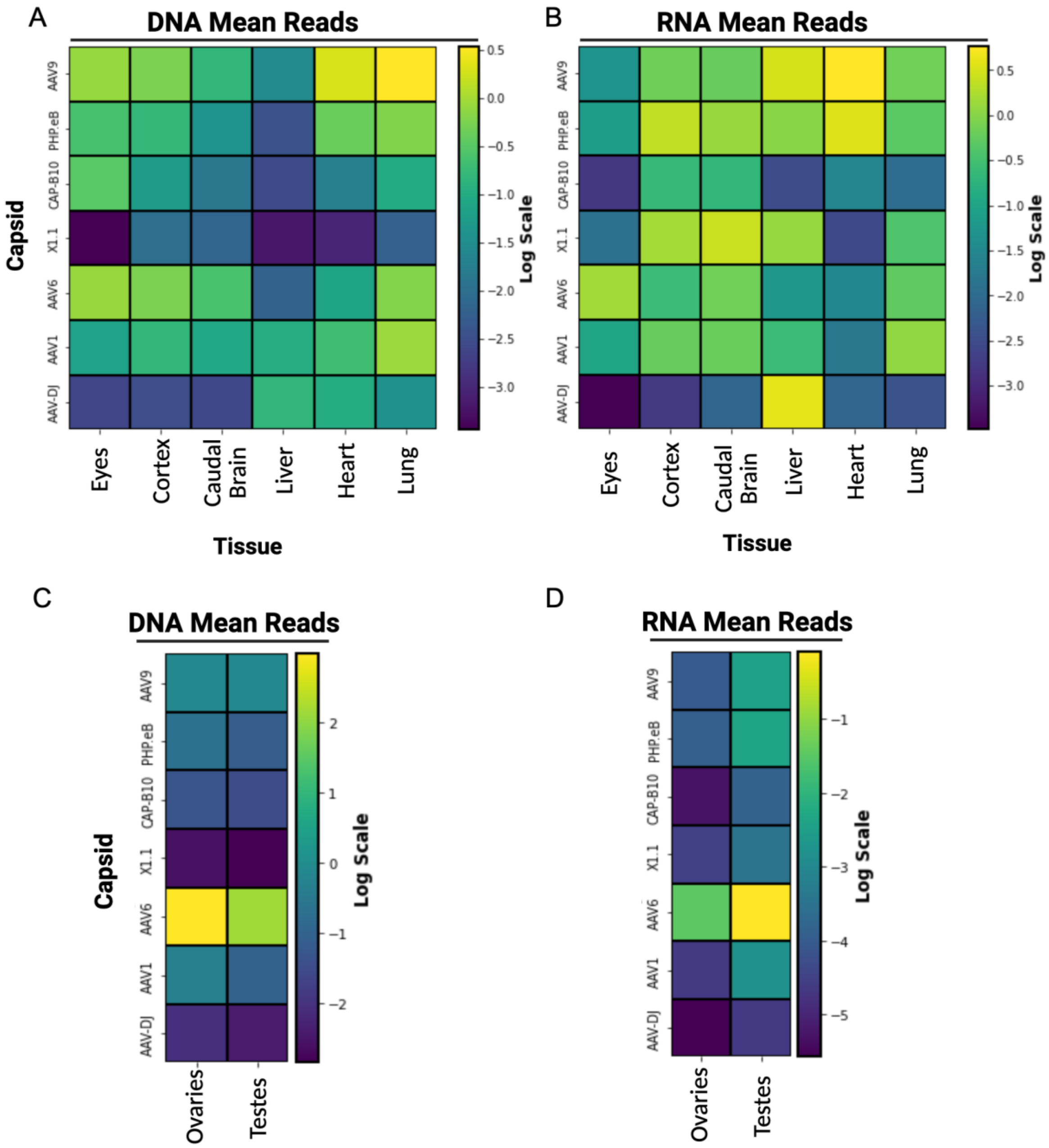

bioRxiv preprint doi: https://doi.org/10.64898/2026.04.28.721509; this version posted May 1, 2026. The copyright holder for this preprint (which was not certified by peer review) is the author/funder, who has granted bioRxiv a license to display the preprint in perpetuity. It is made available under aCC-BY 4.0 International license.

Figure S1: Barcoded AAV capsid screening at E15.5 reveals no enhanced CNS tropism over AAV9.

Eight embryos were injected *in utero* at E15.5 with a pooled barcoded library of seven capsids (AAV9, AAV-DJ, AAV1, AAV6, CAP-B10, PHP.eB, X1.1) at equal concentrations and collected at E18.5. Data were analyzed using linear mixed effects models with tissue, capsid, and normalized reads as fixed effects, and biological replicates as a random effect, with AAV9 set as the reference capsid. **A-B)** Normalized DNA **(A)** and RNA **(B)** abundance of each capsid in cortex, caudal brain, eyes, liver, heart, and lung, relative to AAV9, quantified by NGS. Brain-biased capsids (PHP.eB, CAP-B10, X1.1; tropism established in adult mice) showed no statistically significant enrichment in CNS tissues or depletion in liver (all $p > 0.05$). **C-D)** Normalized DNA **(C)** and RNA **(D)** abundance in gonads (ovaries and testes; $n = 2$ female, $n = 6$ male).

bioRxiv preprint doi: https://doi.org/10.64898/2026.04.28.721509; this version posted May 1, 2026. The copyright holder for this preprint (which was not certified by peer review) is the author/funder, who has granted bioRxiv a license to display the preprint in perpetuity. It is made available under aCC-BY 4.0 International license.

Figure S2: Systemic access via vitelline vein injections at E12.5 and E15.5.

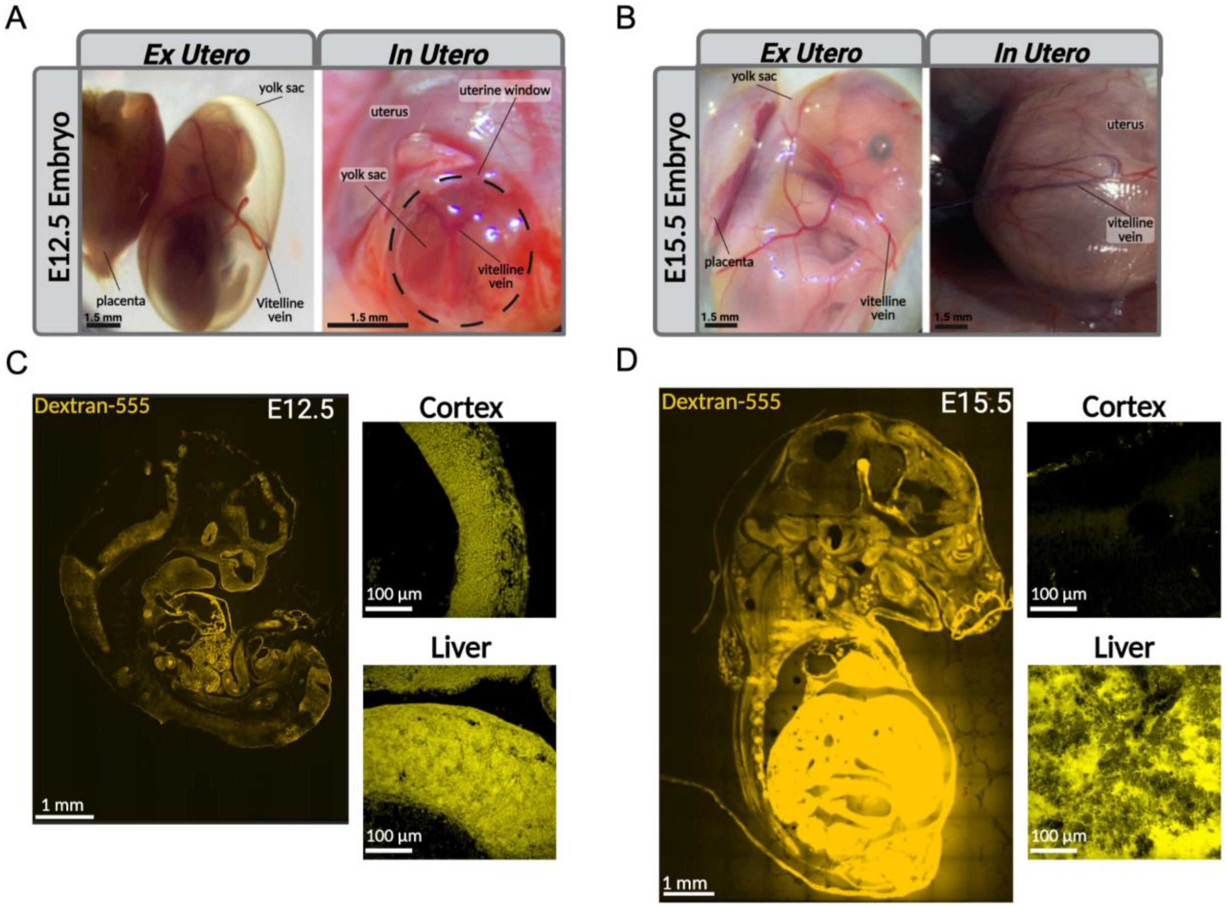

Figure S2: Systemic access via vitelline vein injections at E12.5 and E15.5.

A-B) *In utero* and *ex utero* views of E12.5 (A) and E15.5 (B) embryos, showing yolk sac and uterine anatomy. E12.5 anatomical images are repeated from Figure 1C. **C-D)** Fluorophore-conjugated dextran dye (555 nm, yellow) was injected through the vitelline vein. Embryos were collected within 1 hour of injection. Broad systemic distribution was observed in brain, heart, liver, lungs, and gut. Magnified images of E15.5 cortex and liver highlight dye uptake in organs of interest. Fluorescence intensity was normalized to liver intensity within each embryo (n = 2 embryos per stage)

bioRxiv preprint doi: https://doi.org/10.64898/2026.04.28.721509; this version posted May 1, 2026. The copyright holder for this preprint (which was not certified by peer review) is the author/funder, who has granted bioRxiv a license to display the preprint in perpetuity. It is made available under aCC-BY 4.0 International license.

807 **Figure S3: Age-dependent AAV9 transgene tropism in E15.5- and E12.5-injected embryos.**

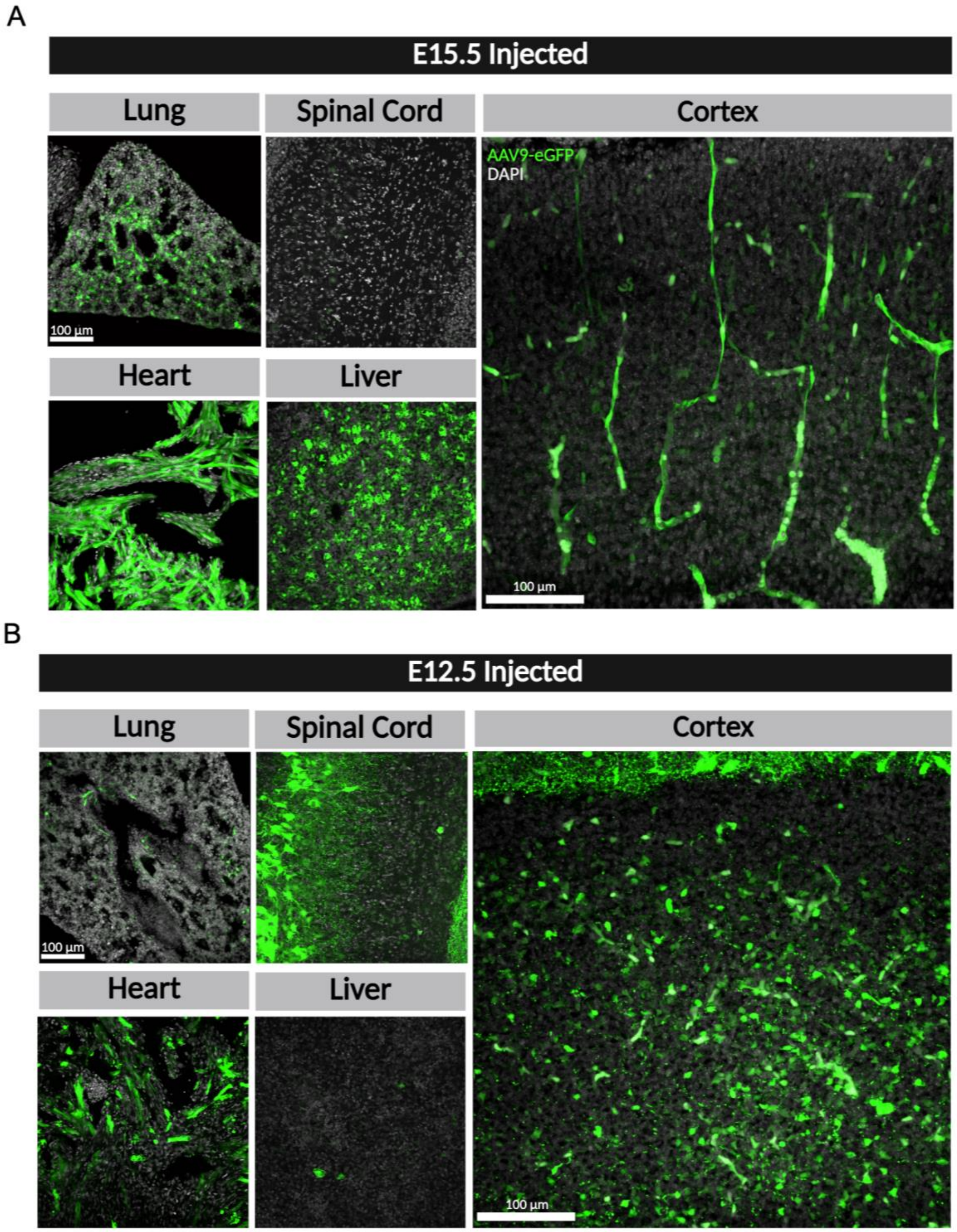

808

809

810

bioRxiv preprint doi: https://doi.org/10.64898/2026.04.28.721509; this version posted May 1, 2026. The copyright holder for this preprint (which was not certified by peer review) is the author/funder, who has granted bioRxiv a license to display the preprint in perpetuity. It is made available under aCC-BY 4.0 International license.

Figure S3: Age-dependent AAV9 transgene tropism in E15.5- and E12.5-injected embryos.

A) Representative images of E18.5 embryos injected at E15.5 with AAV9-eGFP showing transgene expression in heart, liver, and lung with minimal signal in cortex and spinal cord. **B)** Representative images of E18.5 embryos injected at E12.5 with the same AAV9-eGFP construct showing increased expression in cortex and spinal cord and reduced expression in heart, liver, and lung. Images highlight the developmental shift in AAV9 tropism between E12.5 and E15.5 embryos.

bioRxiv preprint doi: https://doi.org/10.64898/2026.04.28.721509; this version posted May 1, 2026. The copyright holder for this preprint (which was not certified by peer review) is the author/funder, who has granted bioRxiv a license to display the preprint in perpetuity. It is made available under aCC-BY 4.0 International license.

Figure S4: AAV-Cre-mediated prenatal induction of reeler cortex.

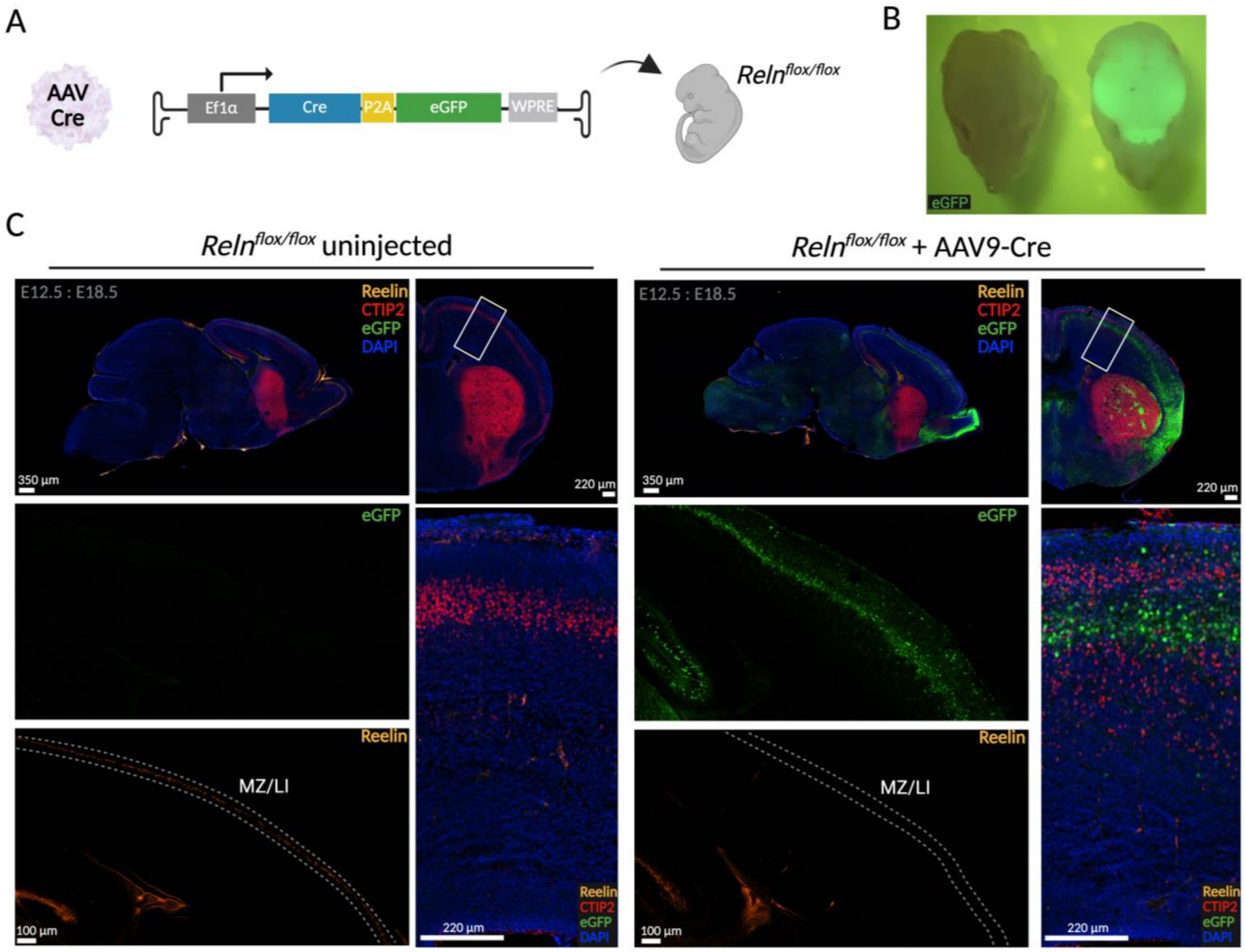

Figure S4: AAV-Cre-mediated prenatal induction of reeler cortex.

A) Schematic of injection of AAV9 carrying a Cre transgene into E12.5 homozygous *Reln*$^{flox/flox}$ embryos. **B)** Brain transduction by Cre-P2A-eGFP transgene at E18.5. Uninjected (left) and injected (right) brains were imaged *in situ* within the skull. **C)** Sagittal E18.5 brain sections showing eGFP and immunostaining for RELN and CTIP2. AAV-Cre injection leads to displacement of deep-layer CTIP2-positive neurons and loss of RELN-positive marginal zone/layer I cells, consistent with prenatal reeler cortex.
